# Exploration of sleep spindles from surgically induced unilaterally deaf adult humans with and without tinnitus

**DOI:** 10.1016/j.cnp.2026.05.001

**Published:** 2026-05-05

**Authors:** MinChul Park, Robin Guillard, Greg A. O’Beirne, Philip A. Bird, Michael R.D. Maslin

**Affiliations:** a University of Canterbury, Private Bag 4800, Christchurch 8140, New Zealand; b Eisdell Moore Centre, Auckland, New Zealand; c GIPSA-Lab, Grenoble INP, CNRS, Université Grenoble Alpes, 38000 Grenoble, France; d Department of Otolaryngology, Head and Neck Surgery and Audiology, Christchurch Hospital, Christchurch, New Zealand; e Department of Surgery, University of Otago, Christchurch, New Zealand

**Keywords:** EEG, Sleep Spindles, Unilateral Deafness, Thalamic Reticular Nucleus, Tinnitus, Neural Plasticity, Noise-Cancellation

## Abstract

**Objective:**

In the neural noise-cancellation mechanism, reduced auditory thalamic reticular nucleus (TRN) activity is thought to result in tinnitus. As a biomarker of TRN activity is sleep spindles, we performed an exploratory study to ask whether individuals with tinnitus would exhibit altered sleep spindles compared with controls.

**Methods:**

Unilaterally deaf adults with tinnitus (UD+T; n = 13), without tinnitus (UD-T; n = 8), and controls without tinnitus or UD (CO; n = 13) were recruited. 2-hour polysomnography nap data were obtained from 64-channels. Spindles were characterised in both frequency and time domains.

**Results:**

We observed no statistically significant differences between our groups in any parameter. However, a dual trend towards lower spindle density (1.8 ± 2.1 spindles/min) and higher spindle amplitude (24.6 ± 9.1 µV) amongst the UD+T group was noted.

**Conclusions:**

A tentative interpretation of the dual trends is the possible altered auditory TRN function associated with tinnitus. Albeit not statistically significant, these exploratory data at least provide a valuable foundation for future studies to probe the effect further. This includes a higher sample size and longer overnight sleep data.

**Significance:**

This is the first study which investigated sleep spindles in UD patients with and without tinnitus.

## Highlights

• Spindles may offer an index of abnormal thalamic reticular nucleus activity.
• Data from unilaterally deaf individuals with and without tinnitus were compared.
• Tinnitus spindle density/amplitude trends were non-significant; replication needed.

## 1 Introduction

Tinnitus can be defined as “the conscious awareness of a tonal or composite noise for which there is no identifiable corresponding external acoustic source” (De Ridder et al., 2021). Typically described by patients colloquially as “ringing in the ears”, tinnitus is a phantom sound with a multifactorial pathophysiology (Adjamian et al., 2014, Adjamian et al., 2009, De Ridder et al., 2021, Ghodratitoostani et al., 2016, Simoes et al., 2021). One common principle thought to underlie most forms of tinnitus is hyperactivity within the central auditory system, triggered by hearing loss, coupled with a reduced ability to “tune out” this noise. This latter element is the focus of the “noise-cancellation” mechanism proposed by Rauschecker et al. (2010), and can help explain why some individuals with hearing loss seem to exhibit tinnitus and others do not.

As with many other explanatory models of tinnitus (Adjamian et al., 2014), the noise-cancellation model presupposes the presence of hearing loss as the trigger. Loss of auditory input due to peripheral deafferentation leads to increased spontaneous neural activity in the auditory brainstem and the medial geniculate nucleus (MGN; Shore et al., 2016). The model also suggests the action of feedback loops involving the limbic system and the thalamic reticular nucleus (TRN; Rauschecker et al., 2010). These loops selectively inhibit or “cancel” components of the neural noise signal. However, in a situation where the feedback loop is absent or dysfunctional, the increased spontaneous activity can pervade to the cortical area leading to tinnitus.

There appears a paucity of direct evidence investigating the TRN and the associated hypothesis about noise-cancellation in humans with tinnitus. According to a recent review (Brinkmann et al., 2021), it seems only one human study has investigated TRN activity in tinnitus patients. Gunbey et al. (2017) used diffusion tensor imaging to compare tissue microstructure integrity of auditory, thalamic and limbic regions (including the TRN) between 36 tinnitus patients and 20 healthy controls. Under the predictions of the noise-cancellation model, individuals with tinnitus should have reduced inhibitory activity from the TRN, which the authors suggested may be reflected by the loss of TRN tissue integrity. Compared to the control group all tinnitus patients showed compromised TRN tissue integrity regardless of the severity of hearing loss. While the study findings were interesting in their own regard, the TRN was one of several other anatomical structures including the amygdala and MGN which also showed the same type of results.

Another way to index TRN function in humans is via sleep spindles. These spindles are characteristic repeating voltage fluctuations that are an EEG hallmark of the N2 stage of non-rapid eye movement (NREM) sleep. The definition provided by the American Academy of Sleep Medicine (AASM) for a sleep spindle in adult humans is “a train of distinct sinusoidal waves with frequency 11 – 16 Hz (most commonly 12 – 14 Hz) with a duration ≥ 0.5 s, usually maximal in amplitude in the central derivations” (Berry et al., 2012, Berry et al., 2020, Iber et al., 2007). Sleep spindles are thought to be generated by the reciprocal synaptic interactions between the TRN and associated thalamocortical neurons, with the TRN behaving as the intra-thalamic pacemaker (Fernandez and Luthi, 2020). Sleep spindles are relatively easily measured from an individual via EEG, with clear and growing evidence linking spindles and key brain functions like learning and memory (Gais et al., 2002), and perceptual disorders like schizophrenia (Ferrarelli et al., 2007).

Despite the relevance of the TRN, the number of studies to have investigated sleep spindles amongst individuals with tinnitus appears to be sparse. Pedemonte et al. (2014) recruited 10 individuals with idiopathic tinnitus and investigated the effects upon overnight sleep EEG of auditory stimulation compared to a quiet control condition. Data from frontal (F3 and F4) and temporal (T3 and T4) sensors were used to document changes in the level of cortical synchronization (i.e. coherence) triggered by the stimulus, which was designed to mimic the participants’ own tinnitus. Data were analysed across a range of oscillatory bands, including the sigma band associated with spindles, and the results revealed generally decreased intra-hemispheric (F3-T3 and F4-T4) and inter-hemispheric (T3-T4) coherences during sound stimulation. On the other hand, Hebert et al. (2011) compared the spectral power in various oscillatory bands, including the sigma band, between 20 tinnitus and control participants, and reported no group differences. Importantly, neither of these studies investigated complimentary spindle data within the time domain, such as spindle amplitude, duration and density (Warby et al., 2014). Furthermore, we know that sleep spindles are not specific to the auditory TRN but rather reflect global TRN function. It is plausible that the conflicting findings might reflect a relatively small effect size caused by spindle data not being specific to the auditory TRN.

One patient group offers an opportunity to further explore TRN biomarkers in humans with tinnitus. These are individuals with surgically induced unilateral deafness (UD) following the removal of a vestibular schwannoma (VS). VS are benign tumours of the 8th cranial nerve sheath (Kim et al., 2001) and are the most frequent type of tumour found in the cerebellopontine angle (Nguyen and de Kanztow, 2019, Pinna et al., 2012). If surgery to remove the VS involves the translabyrinthine approach (Kim et al., 2001, Pinna et al., 2012), the patient will experience complete UD (Leonetti and Marzo, 1999) in a way that is highly consistent from one individual to the next. Postsurgical tinnitus is a frequent symptom, but there is a portion of UD patients who report no tinnitus; around 27% according to Baguley et al. (1992) amongst those without presurgical tinnitus. These combined features offer the unique opportunity to control for common experimental confounds like hearing loss. The abrupt and complete UD caused by surgery tends to result in large scale changes in the central auditory system, thus a relatively large experimental effect size. Some of these effects have previously been documented via auditory evoked potentials of the brainstem and cortex (Maslin et al., 2015, Maslin et al., 2013a, Maslin et al., 2013b, Park et al., 2025a, Park et al., 2025b) but to our knowledge, no prior study has attempted to explore sleep spindle function in this group.

The overarching aim of the present study was therefore to perform an exploratory comparison of sleep spindle patterns amongst individuals with surgically induced UD and their binaurally hearing controls. Our UD cohort was subdivided according to those with associated tinnitus (UD+T) and without tinnitus (UD-T). We have previously reported changes within the central auditory system of the UD participants who participated in the present study, and described how these effects differ according to the presence of tinnitus (Park et al., 2025a, Park et al., 2025b). Here, we hypothesize that individuals in our UD+T group would exhibit altered sleep spindle patterns corresponding with reduced auditory TRN function compared with the other two groups.

## 2 Materials and Methods

### 2.1 Participants

A total of 21 UD participants (9 males, mean ± SD 63.2 ± 9 years; 12 females 64.7 ± 11 years) and matched (age, sex and hearing level) 13 binaurally hearing matched-control (CO) participants (6 males, 70.3 ± 4 years; 7 females 70.3 ± 8 years) were recruited for EEG nap data acquisition. Hearing level of CO participants was matched according to the intact ear (non-surgical) of the UD participants. Inclusion criteria include complete UD post-surgery (as opposed to partial hearing impairment) for the UD group, binaural hearing for the CO group, and medical/physiological capability to undergo EEG nap recording for both groups. Exclusion criteria include previously diagnosed neurological and/or psychiatric disorders (including sleep disorders), use of hearing amplification such as hearing aids and/or cochlear implants and consumption of sleep-altering medications. Preliminary procedures included gathering hearing and health histories, an otoscopic examination, pure tone audiometry (PTA; octave frequencies between 0.25 – 8 kHz), loudness discomfort level (LDL) measurement and transient otoacoustic emissions (TEOAE).

**Fig. 1:**
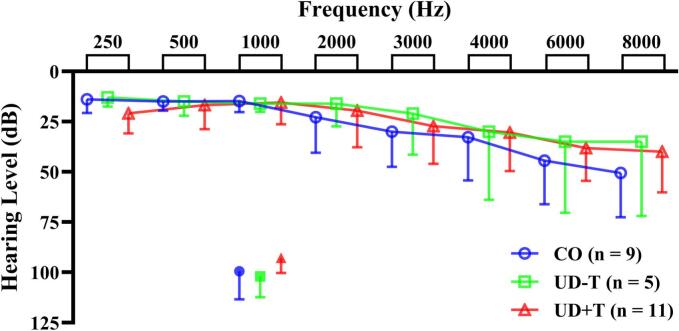
Audiograms for CO (blue), UD-T (green) and UD+T (red) groups. For ease of decipherability, a single error bar is shown (+ 1 SD). Mean + 1 SD loudness discomfort level (filled symbols) at 1 kHz is also shown for each group. (For interpretation of the references to colour in this figure legend, the reader is referred to the web version of this article.)

A Likert scale of tinnitus loudness was used to differentiate UD participants (0 = no tinnitus and 10 = unbearably loud tinnitus), with scores > 1 serving as the boundary. The Tinnitus Handicap Inventory (THI) and Tinnitus Functional Index (TFI) questionnaires were used to capture the severity and functional effects of tinnitus respectively (Fernandez et al., 2022). Finally, the Pittsburgh Sleep Quality Index (PSQI) questionnaire was used to capture the general sleep quality and to control for possible medications participants may be taking for sleeping (Ibanez et al., 2018). A summary of these participants’ characteristics is provided in Fig. 1 and Table 1. All participants gave their written informed consent. All participants gave their written informed consent before participation and the study was approved by the University of Canterbury’s Human Research Ethics Committee (HEC 2021/68/LR-PS) in 2021. The study was carried out between 2021-2024.

**Table 1:** Mean ± SD of participant characteristics. Abbreviations: CO, control; UD-T, unilaterally deaf without tinnitus; UD+T, unilaterally deaf with tinnitus; THI, tinnitus handicap inventory; TFI, tinnitus functional index; PSQI, Pittsburgh Sleep Quality Index.

	**CO (n = 9)**	**UD-T (n = 5)**	**UD+T (n = 11)**
THI	−	2.4 ± 5.4	16.9 ± 11
TFI	−	3.8 ± 8.4	23.3 ± 13.0
Tinnitus loudness (Likert scale 0–10)	−	0.2 ± 0.4	5.5 ± 1.7
Global PSQI	5.4 ± 2.3	5.4 ± 1.7	6.5 ± 2.3

### 2.2 Nap polysomnography (PSG) data acquisition

PSG acquisition was conducted in a sound-treated room in which participants were instructed to lie comfortably on a reclining chair and invited to relax and fall asleep. The recordings began at a time of the participant’s choosing, based on when they would most likely fall asleep (especially for those who usually take daytime naps), but was typically shortly after a midday meal. Each recording session was up to 2 hrs, marking the upper duration of any naps. Two-way audio and visual communication were maintained with the tester throughout.

PSG acquisition involved using a BioSemi ActiveTwo ActiView v.9.02 system with 64-channel EEG electrodes according to the international 10–20 system (Jasper, 1958, Klem et al., 1999) and additional EOG and EMG electrodes according to the AASM guidelines (Berry et al., 2012, Berry et al., 2020, Iber et al., 2007). Contact between the EEG, EOG and EMG electrodes and the scalp were maintained by the application of electrode gel and the electrode offsets were maintained to be within ± 40 mV for each channel. Raw data were sampled at 16.384 kHz with a bandpass filter of DC – 3.2 kHz.

### 2.3 PSG processing

PSG recordings were sampled to 1.024 kHz and a subset of 5 channels (Fpz, Fz, Cz, Pz and Oz) formed the focus of our offline sleep staging analysis. Recording were imported into Brainstorm (Tadel et al., 2011), where data were bandpass filtered (EEG and EOG; 0.3 – 35 Hz and EMG; 5 – 90 Hz; FIR Kaiser type; 0 Hz transition band; 60 dB stopband attenuation) and referenced to the common average. Three personnel (M.P., R.G. and a blinded sleep physiologist) used the AASM guidelines to independently sleep score the recordings, with each person viewing the data in 30 sec epochs (61.1666 pixels/second) and with 50 µV amplitude range (4.6 µV/pixel). Erroneous channels and EEG artifacts including body movements were manually identified and removed from further processing.

For discrete sleep spindle detection, data were broken into epochs (30 s minimum duration) containing N2, N3 and REM sleep phases, as these phases were anticipated to exhibit spindles (Fernandez and Luthi, 2020). Data was further bandpass filtered using Python 10th order (Butterworth 10th order; 0.3 – 30 Hz; Lacourse et al., 2020) and subjected to a previously validated automatic sleep spindle detection algorithm, Slim U-Net trained on MODA (SUMO; Kaulen et al., 2022, MODA = Massive Online Data Annotation; Lacourse et al., 2020), via data from Fpz. The latency and duration of all detected spindles within each epoch were then noted, and if any spindle was detected within a previously labelled artifact, it was removed. Spindles with duration < 0.5 s were also removed.

Once detected and identified, the spindles were then characterized by returning to the original data and analysing power in the sigma spectral band (11 – 16 Hz; FIR Kaiser type; 0 Hz transition band; 60 dB stopband attenuation). Individually extracted spindles were characterized according to the following parameters: duration, intraspindle frequency, symmetry, peak-to-peak amplitude and density (Warby et al., 2014). Spindle parameters were compiled and exported from Brainstorm for statistical analysis.

To characterize the mean power within the σ band for each EEG epoch at Fpz, we notch filtered the raw unfiltered EEG epochs at 50 Hz and computed the power spectral density (PSD; Welch, 1967) over the entire recording length, with a 4 s window and 50% window overlap. Data were normalized (Niso et al., 2019), by scaling power at each frequency relative to the total power over the frequency spectrum of 0.1 – 90 Hz (relative PSD) and log values were computed (Equation (1). To consider the different duration of each EEG epoch, the relative PSD values were further time-normalized according to Equation (2).

We also characterized the mean σ power at Fpz as a ratio of mean power of other neural oscillation bands (Huupponen et al., 2007). The frequency bands were defined as follows (Niso et al., 2019, Tadel et al., 2011); delta (δ; 0.1 – 4 Hz), theta (θ; 5 – 7 Hz), alpha (α; 8 – 12 Hz), beta (β; 13 – 29 Hz), gamma1 (γ1; 30 – 59 Hz) and gamma2 (γ2; 60 – 90 Hz). It would have been mathematically meaningless to perform PSD spectrum normalization (see relative PSD computation in Equation (1)) for each frequency band before computing the ratio with respect to the σ band, as the denominator of Equation (1) would cancel out as a common factor during the computation process. Therefore, the ratio was calculated using the logarithm of absolute PSD values for each frequency band. Additionally, the time normalisation process described in Equation (2) was applied to these ratio computations. The complete equation of this computation is shown in Equation (3).

(1) $$Relative\;PSD\;\left(f\right)=\;10\;\times \log\left(\frac{Absolute\;PSD\;(f)}{\sum_{n}\left[PSD\;{(f}_{n}\right)]}\right)$$

**Equation** (1). Mathematical definition of relative power spectrum density (PSD) where f is the frequency or frequency band of interest (e.g. σ band) and f_n_ are the frequency values across the entire values of the absolute PSD which in this case was between 0.1 – 90 Hz.

(2) $$Time\;normalised\;relative\;PSD\;\left(f\right)=\;\frac{\sum_{1}^{n}\left[Relative{\left(PSD\right)}_{n}\;\times \;Time{\left(EEG\right)}_{n}\right]}{\sum_{1}^{n}\left[Time{\left(EEG\right)}_{n}\right]}$$

**Equation** (2). Time normalisation of relative PSD values by incorporating the time duration of each EEG epoch. The numerator is the sum of relative PSD multiplied by time duration of EEG epoch from the first epoch to the nth epoch. The denominator is the time duration sum of all EEG epochs.

(3) $$Time\;normalised\;PSD\;ratio\left(\frac{\sigma }{f}\right)=\;\frac{\sum_{1}^{n}\left[{\left(\frac{\log(Absolute\;PSD\;\left(\sigma_{mean}\right))}{\log(Absolute\;PSD\;\left(f_{mean}\right))}\right)}_{n}\;\times \;Time{\left(EEG\right)}_{n}\right]}{\sum_{1}^{n}\left[Time{\left(EEG\right)}_{n}\right]}$$

**Equation** (3). Time normalisation of PSD ratios. The numerator is the sum of PSD ratio multiplied by time duration of EEG epoch from the first epoch to the nth epoch. The denominator is the time duration sum of all EEG epochs. σ = sigma band. f = neural oscillation band.

### 2.4 Statistical analysis

Group differences in σ power, ratio and spindle density were analysed via an ordinary one-way analysis of variance (ANOVA). To handle a varying number of data points (i.e. number of sleep spindles detected) given from each participant, a nested one-way ANOVA was used to analyse spindle duration, frequency, symmetry and amplitude. Both ANOVA findings were further analysed using post-hoc Tukey’s multiple comparisons test where appropriate.

## 3 Results

### 3.1 Sleep spindle characterisation

**Fig. 2:**
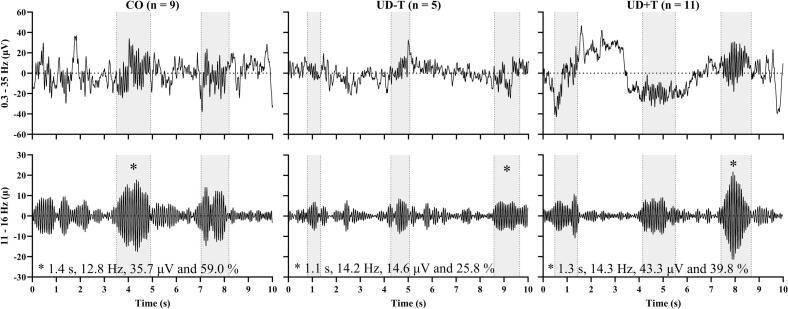
Examples of Fpz spindles detected by SUMO (shaded in grey) from each group. Left, middle and right for CO (n = 9), UD-T (n = 5) and UD+T (n = 11) groups respectively. Top and bottom row for 0.3 – 35 and 11 – 16 Hz filtered recordings respectively. Spindle duration, frequency, amplitude and symmetry values of an example detected spindle are specified (marked by *).

**Fig. 3:**
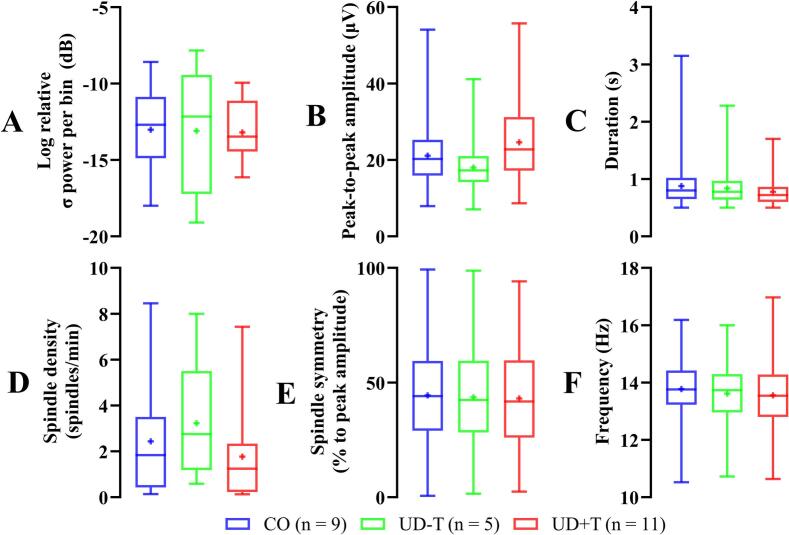
Summary of Fpz σ power and sleep spindle characteristics between groups. A: σ power, B: amplitude, C: Duration, D: Density, E: Symmetry and F: Frequency. Blue, green and red denotes the CO (n = 9), UD-T (n = 5) and UD+T (n = 11) groups respectively. + = mean. (For interpretation of the references to colour in this figure legend, the reader is referred to the web version of this article.)

Of the 21 participants with UD, N2 stage data was gathered from 16 participants during the nap cycle, and similarly from 9 of the 13 controls. Descriptive data from the sleep staging analysis is offered in Table 2. Using data from these participants, the SUMO automatic sleep spindle detection algorithm identified a total of 475, 260 and 221 spindles from our CO, UD-T and UD+T groups respectively. Examples of spindles detected by SUMO are shown in Fig. 2 while a series of box-and-whisker plots summarizing the spindle parameters are shown in Fig. 3. Qualitative examination of Fig. 3 shows mean spindle duration, symmetry and frequency were similar between groups (Fig. 3C, E and F respectively). Spindles from the UD+T group had the highest mean amplitude (Fig. 3B) and the lowest mean spindle density (Fig. 3D) out of the three groups. However, none of the statistical comparisons revealed a statistically significant difference associated with these trends (Table 3).

**Table 2:** Duration of sleep stages including N1, non-rapid eye movement stage 1; N2, non-rapid eye movement stage 2; N3, non-rapid eye movement stage 3; REM, rapid eye movement.

**Duration (mins)**	**CO (n = 9)**	**UD-T (n = 5)**	**UD+T (n = 11)**
N1	15.7 ± 7.6	12.9 ± 4.2	14.1 ± 10.3
N2	23.8 ± 11.4	22.1 ± 21.4	16.0 ± 13.8
N3	4 ± 7.9	0	3.8 ± 5.5
REM	0	6.9 ± 15.4	0
Total Sleep	43.5 ± 15.3	41.9 ± 39.1	34.0 ± 19.7

**Table 3:** Ordinary (σ power, ratio and spindle density) and nested (spindle amplitude, duration, symmetry and frequency) one-way ANOVA results summary, with statistical effect size estimates (^a^ partial eta-squared statistic).

**Data**	**F-statistic (df)**	**P-value**	**Effect size ^a^**
σ power	F (2, 22) = 0.009	0.991	0.001
σ/δ ratio	F (2, 22) = 0.005	0.995	0.000
σ/θ ratio	F (2, 22) = 0.723	0.497	0.062
σ/α ratio	F (2, 22) = 0.735	0.491	0.063
σ/β ratio	F (2, 22) = 0.705	0.505	0.060
σ/γ1 ratio	F (2, 22) = 1.109	0.348	0.092
σ/γ2 ratio	F (2, 22) = 2.540	0.102	0.188
Density	F (2, 22) = 0.629	0.543	0.054
Amplitude	F (2, 22) = 0.777	0.472	0.066
Duration	F (2, 22) = 1.041	0.370	0.086
Symmetry	F (2, 22) = 0.328	0.724	0.029
Frequency	F (2, 22) = 0.207	0.815	0.018

### 3.2 Sigma band power and ratio

**Fig. 4:**
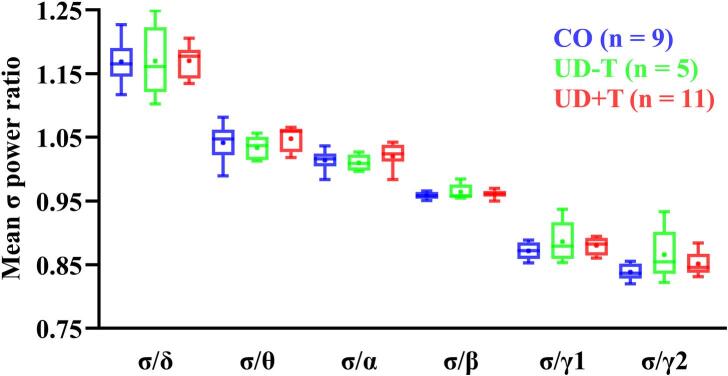
Mean Fpz σ power as a ratio to six other EEG frequency bands. Blue, green and red denotes the CO (n = 9), UD-T (n = 5) and UD+T (n = 11) groups respectively. δ; 0.1 – 4 Hz, θ; 5 – 7 Hz, α; 8 – 12 Hz, β; 13 – 29 Hz, γ1; 30 – 59 Hz and γ2; 60 – 90 Hz. + = mean. (For interpretation of the references to colour in this figure legend, the reader is referred to the web version of this article.)

The σ power is shown in Fig. 3A, and the same data are replotted as a ratio of adjacent oscillatory band powers (Fig. 4). The overall band power (and therefore ratios) falls inversely to the frequency. However, our findings do not suggest any systematic trends for differences in band power data either between the two UD groups or compared with controls and indeed, none of the statistical comparisons revealed any significant effects (Table 3).

## 4 Discussion

### 4.1 Study summary and key results

The objective of this exploratory study was to characterize sleep spindles in UD participants with and without tinnitus and their binaurally hearing controls. The overarching aim was to probe auditory TRN activity associated with tinnitus, with spindles being a biomarker of TRN function. As far as we are aware, this is the first study that has attempted such an exploration using the surgically induced UD population. We have quantified sleep spindle activity within the frequency domain via computing the σ spectral power and within the time domain via identifying discrete spindles and computing a range of spindle parameters. Although some potentially interesting trends emerged, contrary to our hypotheses the results revealed no statistically significant differences in σ power and sleep spindle parameters between individuals with UD and associated tinnitus, or UD without tinnitus, and their binaurally hearing controls. Although tentative, some of our observations may warrant further investigation and in that case, the present data may at least help inform future study design, for instance with respect to statistical power and sample size estimates.

### 4.2 Sigma band power and sleep spindle characteristics

One method to quantify sleep spindles is to compute the spectral power of the obtained EEG within the limits of the spindle frequencies, called the σ band (i.e. frequency domain analysis). This type of quantification considers spindle activity as a wide category of oscillations from its background EEG form to its transient form (the typical sleep spindle) regardless of its exact occurrence as discrete events in time (Dumermuth et al., 1972, Kubicki and Herrmann, 1996). We hypothesized that the UD+T group would show altered σ power compared to the other two groups due to the supposed dysfunction of the TRN. However, our results did not reveal any such alteration.

Another analytical approach is to identify discrete transient spindle events in time as they naturally occur, and to subsequently quantify the properties of these events. Spindles can be categorized into two types based on their oscillation frequency (Cox et al., 2017, Mölle et al., 2011). Slow spindles (< 12.5 Hz) are associated with N3 sleep while fast spindles (> 12.5 Hz) are associated with N2 sleep (Fernandez and Luthi, 2020). Our sleep staging analysis identified spindles occurring primarily in N2 and consistent with this, the mean spindle frequency from each of our three groups were in the fast category. We predicted that if the UD+T group exhibited some form of dysfunctional TRN, then the spindles from individuals in this group would show altered characteristics in comparison to the UD-T and CO groups. However, we observed no statistically significant differences between groups at any spindle parameter.

Previously, amongst the same participants, we have noted data from cortical auditory evoked potentials that suggests reduced adaptation in response to signals presented in noise amongst the UD+T group (Park et al., 2025a), with inhibitory feedback thought to drive the adaptation under normal circumstances. In this context, some trends within the present findings may be worth highlighting, although there were no statistically significant group differences. In particular, we noted how spindle density was somewhat lower while spindle amplitude was somewhat higher in the UD+T group. A related pattern (but in the opposite direction) has been previously observed in patients with some psychotic disorders, where spindle density within the clinical population was higher than the controls, yet spindle amplitude was lower (Manoach et al., 2014). Supposing that the present trend is a true effect, one possible explanation is impaired auditory TRN function amongst UD+T individuals, reflected by fewer spindles generated per unit time. It may be that with a lower density then each spindle’s amplitude increases in some compensatory fashion (Fernandez and Luthi, 2020). In any case, it may be interesting to determine in the future whether the trends we report here are meaningful.

Our exploratory approach invites several reflections. For one thing, our hypothesis is focused on TRN activity in relation to the auditory system because participants had tinnitus and does not necessarily extend to non-auditory TRN function. On the other hand, our spindle analyses are not thought to be specific to the auditory TRN neurons, as presumably non-auditory TRN sources contribute to the observed spindles. The effect of this would be to reduce our data specificity and reduce statistical power. Another reflection is the relevance of different durations of N2 sleep in each of our participants, with some not napping for long enough (or at all) to reach N2 for long enough periods. In turn, this influences the number of detected spindles in the three groups in a way that would also influence statistical power: The UD+T group, who also had the highest PSQI scores, showed the shortest duration of N2 sleep accompanied by lowest number of detected spindles and spindle density (which would have lowered the overall σ power) while concurrently, also showing the highest spindle amplitude, which may have the opposite effect (elevating the overall σ power).

It is known that sleep spindles are highly sensitive to circadian rhythm (Dijk and Czeisler, 1995) and are strongest in power during habitual sleep (Fernandez and Luthi, 2020). Although our data partially control for circadian rhythm variations by initiating the PSG recording at a similar time of day in each participant, we lack PSG data (Goldschmied et al., 2021, Lechat et al., 2021, Wamsley et al., 2012, Warby et al., 2014). It seems overall that overnight PSG data would at least partially address both of these reflections, and this therefore forms one recommendation emerging from our findings for any future investigation. The additional overnight PSG data may be used to validate the current findings.

All components of sleep are affected by aging and sleep spindles are no exception. Sleep shows reduced functionality with decrease in sleep spindle density, amplitude and duration with aging (Fernandez and Luthi, 2020). In our study, the CO group was, on average, older than both UD groups and may have contributed to the study results. However, this is unlikely to be the case as spindle amplitude and density were higher in the UD+T and UD-T groups respectively. If age was a confounding factor, its effects would have affected both UD groups similarly. The current diverging dual trends dependent on the presence of tinnitus seem to suggest otherwise. Furthermore, each control and UD participant were matched according to their hearing levels which further suggests that age-related hearing loss may not be a contributing factor.

A final reflection in our approach is avoidance of the M1 and M2 sensor positions in our montage, even though the mastoid processes serve as the standard reference sites recommended by AASM (Berry et al., 2020). The reasons for not using M1 and M2 were twofold: discomfort reported by some participants but more importantly, anatomical differences as a consequence of translabyrinthine surgery (Leonetti and Marzo, 1999). Mastoidectomy is a surgical step in the translabyrinthine surgical approach the UD participants went through for their unilateral VS removal. That is, as a consequence of VS removal, the UD participants had anatomically different mastoid areas: a physically intact non-surgical side and the ablated surgery side.

## 5 Conclusions and future directions

This was the first study that examined sleep spindles from individuals with surgically induced UD with and without tinnitus as a way to probe the potential role of the TRN in the noise-cancellation mechanism of tinnitus. There was no statistical difference in the relative σ spectral power, σ power ratios, or time domain sleep spindle parameters between the three study groups. Nevertheless, notable trends for reduced spindle density and increased amplitude were found amongst individuals with UD and tinnitus. The results do not offer any basis to reject our null hypothesis. However, it gives a reminder that these results were set in the context of a nap study with relatively small sample size. The results provide several recommendations for future studies (e.g. higher sample size and acquisition of overnight sleep data) looking to shed further light on the possible association between TRN and tinnitus generation in humans.

## Funding

This research was funded by the University of Canterbury’s Doctoral Scholarship.

## Declaration of competing interest

The authors declare that they have no known competing financial interests or personal relationships that could have appeared to influence the work reported in this paper.
